# Microwave-Assisted Extraction of *Pleurotus* Mushrooms Cultivated on ‘Nero di Troia’ Grape Pomace and Evaluation of the Antioxidant and Antiacetylcholinesterase Activities

**DOI:** 10.3390/jof11110783

**Published:** 2025-10-30

**Authors:** Gaetano Balenzano, Anna Spagnoletta, Giovanni Lentini, Gennaro Brunetti, Francesco De Mastro, Mariagrazia Rullo, Leonardo Pisani, Fortunato Cirlincione, Maria Letizia Gargano, Maria Maddalena Cavalluzzi

**Affiliations:** 1 Department of Soil, Plant and Food Science (Di.S.S.P.A.), Campus E. Quagliarello, University of Bari Aldo Moro, Via Edoardo Orabona 4, 70125 Bari, Italy; gaetano.balenzano@uniba.it (G.B.); gennaro.brunetti@uniba.it (G.B.); francesco.demastro@uniba.it (F.D.M.); 2 Division Sustainable Agrifood Systems (SSPT-AGROS-BIOEC), ENEA-Research Center Trisaia, S.S. Jonica 106, km 419.5, 75026 Rotondella, Italy; anna.spagnoletta@enea.it; 3 Department of Pharmacy–Pharmaceutical Sciences, University of Bari Aldo Moro, 70125 Bari, Italy; giovanni.lentini@uniba.it (G.L.); mariagrazia.rullo@uniba.it (M.R.); leonardo.pisani@uniba.it (L.P.); mariamaddalena.cavalluzzi@uniba.it (M.M.C.)

**Keywords:** sustainability, circular economy, Alzheimer’s disease, MAE, GC-MS, *eryngii*, *ostreatus*, biological activity, medicinal mushroom

## Abstract

The sustainable management of winery residues could represent a cornerstone for promoting environmental and economic sustainability from a circular economy perspective. In this context, our study aimed to evaluate *Vitis vinifera* L. ‘Nero di Troia’ cultivar grape pomace as a valuable waste product for the cultivation of *Pleurotus* mushroom, in comparison with traditional wheat straw-based cultivation. Mushroom extracts were prepared through the eco-friendly microwave-assisted extraction technique, using green solvents with different polarity degrees. Total protein content, total polyphenol content, and antioxidant activity (FRAP and DPPH assays) were assessed for the water and EtOH hydrophilic extracts. Grape pomace often gave higher values than wheat straw, especially for the *P. eryngii* var. *eryngii* water extract protein content, which was 3.5-fold higher (0.68 ± 0.14 mg BSA/mL and 0.192 ± 0.025 mg BSA/mL, respectively). The ethyl acetate extracts of both mushroom species gave biologically relevant results in terms of inhibiting activity against acetylcholinesterase, an enzyme involved in the pathogenesis of Alzheimer’s disease (50% inhibitory activity at concentrations ≤ 1.5 mg/mL), thus paving the way for more in-depth investigation. The extract’s metabolic profile was investigated through GC-MS analysis. The results show that incorporating grape pomace into mushroom production represents a concrete step toward more sustainable biotechnological processes.

## 1. Introduction

The concept of the circular economy (CE) aims to close the loop of material flows by converting waste into resources, thereby reducing environmental impacts and promoting sustainable development [1]. In this context, grape pomace, the most considerable by-product of winemaking, represents a valuable resource for advancing sustainability efforts in wineries, both financially and ecologically, and it is a notable example of CE practices within winemaking by-products. It is estimated that more than 79 million tons of grapes are processed annually, generating grape pomace in amounts of approximately 30% of the total volume [2]. This by-product mainly consists of grape skins, pulp, seeds, and stems generated through fermentation and/or mechanical extraction. Its composition can differ greatly depending on factors like the grape cultivar (cv.), agricultural practices, and environmental influences [3]. Grape pomace contains abundant dietary fiber, remaining sugars, fats, proteins, phenolic substances, and various other important bioactive components [4].

An additional emerging sector with significant potential for the valorization of agricultural waste is mushroom cultivation. Fungi play a crucial role in the natural decomposition of lignocellulosic biomass through the secretion of a wide variety of lignocellulose-degrading enzymes. Numerous fungal species secrete substantial quantities of enzymes that function cooperatively in their surrounding environment [5]. The transformation of lignocellulosic biomass through fungal processes offers significant potential for advancing circular bioeconomy strategies, supporting efficient resource use and minimizing waste generation [6]. Fungi (especially species from the Basidiomycota and certain Ascomycota) have been utilized for medicinal purposes for centuries. Their fruiting bodies, cultivated mycelium, and fermentation broths are rich in diverse bioactive substances such as polysaccharides and lectins, which have shown beneficial effects on health, including antioxidant, antiviral, and anticancer activities [7,8].

Due to their strong biodegradative abilities, high nutritional content, and extensive European distribution, species of the genus *Pleurotus* (Fr.) P. Kumm. represent a promising strategy for minimizing the environmental burden of agri-food waste while simultaneously converting it into valuable edible products [9]. *Pleurotus* mushroom production represents approximately 25% of the total global cultivated mushroom. Their fruiting bodies offer a substantial source of protein, dietary fiber, carbohydrates, essential vitamins, and minerals [10]. Conventional substrates used for fungal growth typically consist of agricultural by-products, wood industry residues, and food processing by-products [11]. Recent research, however, has highlighted the potential of grape pomace as a substrate for fungal cultivation due to its rich lignocellulosic matrix, moderate sugar, and phenolic content [12,13].

Within this framework, attention has turned toward optimizing the recovery of bioactive compounds from mushroom biomass grown on grape residues, with the extraction methods playing a pivotal role. Conventional techniques (hot water, solvent extraction) are often time-consuming, inefficient, and environmentally burdensome due to solvent use and high energy demand [14]. Therefore, green and innovative extraction techniques capable of enhancing extraction yield, reducing solvent consumption, and lowering processing times have been developed in recent decades [15].

Between these, based on our previous experience, microwave-assisted extraction (MAE) was selected for its potential to enhance extraction efficiency and reduce processing time and solvent use [16].

Microwaves are electromagnetic waves with wavelengths between 0.001 and 1 m and frequencies in the 30–300 GHz range, which can influence both the activity and the structural features of biopolymers. In MAE, the rise in temperature and pressure weakens the interaction between solutes and the sample matrix. The extraction solvent then permeates the matrix, dissolving the target compounds. Because microwaves generate heat directly inside the material through dipole rotation and ionic conduction, bioactive molecules can be efficiently released and recovered [17].

The present study started following the cultivation of *Pleurotus ostreatus* (Jacq.) P. Kumm. and *Pleurotus eryngii* (DC.) Quél. var. *eryngii* using grape pomace (*Vitis vinifera* L.) of cv. ‘Nero di Troia’ as a substrate or in combination with wheat straw conventionally used as a substrate for *Pleurotus* mushroom cultivation [18]. To valorize fungal material produced using agro-industrial byproducts, bioactive extracts were obtained through the green and innovative MAE procedure. Antioxidant activity was evaluated for extracts obtained using water and absolute ethanol as extraction solvents. The inhibiting activity against AChE, an enzyme involved in the pathogenesis of Alzheimer’s disease (AD), was evaluated for the more lipophilic extracts obtained with ethyl acetate. The chemical profiles of the mushroom extracts were also assessed through gas chromatography–mass spectrometry (GC-MS) analysis.

## 2. Materials and Methods

### 2.1. Origin and Physicochemical Characterization of the Substrate

The grape pomace (P) derived from the winemaking of cv. ‘Nero di Troia’, a red cultivar typical of Southern Italy, was provided by Trulli il Castagno snc (Martina Franca, Italy). Wheat straw (S) was provided by the Department of Soil, Plant and Food Sciences (Di.S.S.P.A.), University of Bari Aldo Moro (Italy). Before the analyses, the samples were air-dried and ground to 0.5 mm. Moisture content, expressed as a percentage of the initial weight, was determined by drying the samples at 105 °C overnight, while ash content was measured after overnight incineration at 550 °C in a muffle furnace [19]. The pH was measured in sample/water extracts (1:20 *w*/*v*), whereas the contents of different carbon fractions (TOC 400, ROC, and TIC) and total nitrogen (TN) were determined using the soli TOC^®^ cube elemental analyzer (Elementar, Lomazzo, Como, Italy) Table 1 in Ref. [20].

For the determination of total heavy metals (Cd, Cr, Cu, Ni, Pb, and Zn) and base cations (Ca, K, Mg, and Na), samples were subjected to microwave-assisted acid digestion using a CEM DISCOVER SP-D (CEM corporation, Charlotte, NC, USA) system with appropriate vessels. Approximately 0.3 g of sample was digested with a suprapure HNO_3_:HCl mixture (5:1, *v*/*v*). After digestion, the solutions were cooled, diluted to 25 mL with Milli-Q reagent-grade water in volumetric flasks, filtered through Whatman No. 42 paper, and subsequently analyzed using an ICP-OES iCAP 6300 inductively coupled plasma optical emission spectrometer (Thermo Fisher Scientific Inc., Waltham, MA, USA) Table 2 in Ref. [21]. Analysis of crude fiber (CF), acid detergent fiber (ADF), neutral detergent fiber (NDF) and acid detergent lignin (ADL) were determined by analyzing 0.5–0.55 g of samples using an ANKOM 200 automated system with specialized filter bags (ANKOM Technology, Macedon, NY, USA) [22]. The values measured in this study are consistent with the ranges reported by other researchers [23,24,25].

### 2.2. Fungal Strain

The experimental trials included the cultivation of two distinct mushroom species: *Pleurotus ostreatus* BAF7 strain (PO) and *Pleurotus eryngii* var. *eryngii* BAF6 strain (PE). Both fungal species were deposited in the Mycoteca of the Department of Agricultural, Food and Forest Sciences (SAAF) (University of Palermo, Italy). The samples were supplied by the Department (SAAF) and stored at 4–8 °C on Potato Dextrose Agar (Thermo Fisher Scientific Inc., Waltham, MA, USA) in test tubes and periodically transferred until further use.

### 2.3. Substrate Preparation and Mushroom Cultivation

A piece of purified mycelium from PO and PE was utilized to prepare spawn by inoculating sorghum seeds. These seeds had been pre-soaked in distilled water, transferred into 1 L jars, and sterilized at 121 °C for 20 min. The substrates were prepared in jars with a maximum capacity of 1 L. Three different substrates were used for the cultivation of both fungal species: wheat straw (S); grape pomace (P); and a 1:1 (*w*/*w*) mixture (M) (on a dry weight basis) of wheat straw and grape pomace. The experiment was carried out in three replicates for each substrate used and both fungal species. Once the substrate was fully colonized by the mycelium and deemed suitable for basidiome production, it was conducted in a greenhouse facility of the department. Once harvested, the fruiting bodies were sliced and dehydrated using a Valla air dryer (Borgotaro, Parma, Italy), ground with a blade grinding device MB 950S MICROTRON (Kinematica AG, Malters, Switzerland) and stored at 4 °C in vacuum-sealed bags until further use.

### 2.4. Microwave-Assisted Extraction (MAE)

Microwave-assisted extraction (MAE) of the mushroom powder was performed using a CEM Discover Bench Mate (CEM corporation, Charlotte, NC, USA) microwave reactor equipped with Synergy software (SKU 909100), following the method described by [26], with slight modifications. Briefly, a 1:30 (*w*/*v*) solid-to-solvent ratio was used (e.g., 33.5 mg of PO/PE powder in 1.0 mL of solvent) and irradiated at 80 °C for 5 min under closed-system conditions with continuous stirring. Three solvents with different polarities were used: water (W), absolute ethanol (E) and ethyl acetate (A). The microwave power ranged from 30 to 100 Watt, according to the solvent used; specifically, 30 Watt was applied for water and ethanolic extraction, and 100 Watt for extracts with ethyl acetate. After filtration, the extracts were centrifuged at 8000 rpm for 10 min, and the supernatants were collected. The solvent was subsequently removed under reduced pressure using a rotary evaporator or lyophilized, and the resulting solid residue was stored at −20 °C until further analysis.

### 2.5. Antioxidant Properties

#### 2.5.1. Total Protein Content

The quantification of total proteins was carried out employing a modified Bradford assay, according to the procedure outlined by Baldacchino et al. [27]. In brief, 10 µL of suitably diluted sample extract was dispensed into each well of a 96-well microplate, to which 200 µL of pre-diluted Bradford reagent was subsequently added. The microplate was then shaken for 30 s using the built-in shaker of a microplate reader and incubated in the dark at ambient temperature for 10 min. The absorbance was recorded at 595 nm employing a Multiskan Go Spectrophotometer (Thermo Fisher Scientific Inc., Waltham, MA, USA). Protein concentration was determined based on a standard calibration curve prepared with bovine serum albumin (BSA).

#### 2.5.2. Total Polyphenol Content (TPC)

Total polyphenol content (TPC) was assessed in the sample extracts using a modified version of the Folin–Ciocalteu method, as previously described [28]. To remove protein interference, aliquots of each extract were first treated with trichloroacetic acid (final concentration, 15%) to precipitate proteins [29]. Subsequently, 20 µL of the appropriately diluted protein-free sample was added to a 96-well microplate along with 100 µL of diluted Folin–Ciocalteu reagent (1:10 dilution). After a 5 min incubation at room temperature, 80 µL of a 7.5% sodium carbonate (Na_2_CO_3_) solution was added to each well. The reaction mixtures were then incubated in the dark for 60 min, and absorbance was measured at 760 nm using a Multiskan Go Spectrophotometer (Thermo Fisher Scientific Inc., Waltham, MA, USA). TPC values were calculated using a standard calibration curve of gallic acid and expressed as milligrams of gallic acid equivalents (mg GAE) per 100 g of sample.

#### 2.5.3. Ferric Reducing Antioxidant Power (FRAP)

The ferric reducing antioxidant power (FRAP) of the extracts was assessed according to the method described by Sousa-Santos et al. [30] with minor modifications. Ascorbic acid was used as the calibration standard. Briefly, 20 µL of each extract were mixed with 280 µL of freshly prepared FRAP reagent, consisting of 0.3 M acetate buffer (pH 3.6), 10 mM 2,4,6-tris(2-pyridyl)-S-triazine (TPTZ) dissolved in 40 mM HCl, and 20 mM ferric chloride (FeCl_3_), combined in a 10:1:1 (*v*/*v*/*v*) ratio. The reaction mixture was incubated at room temperature for 30 min, after which absorbance was measured at 593 nm using a Multiskan Go Spectrophotometer (Thermo Fisher Scientific Inc., Waltham, MA, USA). FRAP values were expressed as milligrams of ascorbic acid equivalents per 100 g of sample (mg AAE/100 g sample).

#### 2.5.4. DPPH Radical Scavenging Activity

The antioxidant capacity of the extracts was evaluated using the 1,1-diphenyl-2-picrylhydrazyl (DPPH) radical scavenging assay, following the method described by Hamed et al. [31] with minor modifications. Trolox was employed as the reference antioxidant standard. In brief, 30 µL of each were combined with 20 µL of distilled water and subsequently mixed with 200 µL of a DPPH solution (60 µg/mL in methanol) in a 96-well microplate. The mixtures were incubated in the dark for 30 min at 25 °C. Following incubation, absorbance was measured at 515 nm using a Multiskan Go Spectrophotometer (Thermo Fisher Scientific Inc., Waltham, MA, USA). The results were expressed as milligrams of Trolox equivalents per 100 g of sample (mg Trolox Eq/100 g sample).

### 2.6. GC-MS Analysis

Secondary metabolites were analyzed using an HP–5MS capillary column (30 m × 0.25 mm, i.d. 0.25 µm) (Agilent, Santa Clara, CA, USA). In total, 1 µL sample was injected using a split ratio of 1:100. The column was held at 70 °C for 3 min after injection, the temperature programmed at 3 °C/min to 280 °C and held for 20 min more. Helium was used as carrier gas, at a constant column flow rate of 1.5 mL/min. The injector temperature was 250 °C, and the detector temperature was 230 °C. The mass spectrometer was operated at 70 eV with a mass range from 30 to 400 atomic mass units (AMU). Secondary metabolites were identified by comparing their retention times and mass spectra with those available in the National Institute of Standards and Technology (NIST) Mass Spectral Library Version NIST 11 and/or reported in data from the NIST standard reference database (NIST chemistry webbook) and in the literature. Only compounds with a NIST similarity of at least 80% were considered. Results were expressed as the individual relative percentage of each secondary metabolite present in the sample [26].

### 2.7. Evaluation of Acetylcholinesterase Inhibitory Activity 

Human acetylcholinesterase, substrates, and reagents were from Sigma-Aldrich (Milan, Italy). As reported elsewhere [32], experiments were run in 96-well plates from Greiner Bio-One (Kremsmenster, Austria) by using an Infinite M1000 Pro multiplate reader (Tecan, Cernusco sul Naviglio, Milan, Italy). The inhibition of AChE was determined by following Ellman’s spectrophotometric assay in transparent, flat-bottom plates. Incubations were performed in triplicate and results were expressed as the mean ± S.D. from 3 independent experiments.

### 2.8. Statistical Analysis

Statistical analyses were performed using GraphPad Prism version 5.0 (GraphPad Software, San Diego, CA, USA). The normality of data distribution was assessed using the Shapiro–Wilk test. All results are presented as means ± standard deviation (S.D.) from at least three independent experiments. Differences among groups were evaluated by one-way analysis of variance (ANOVA), followed by Tukey–Kramer’s post hoc test for multiple comparisons. *p*-values < 0.05 were considered statistically significant. Values sharing the same letter are not significantly different at α = 0.05.

## 3. Results and Discussion

### 3.1. Substrate Analyses

Since organic wastes vary widely in origin, physical characteristics, and chemical composition, dedicated research is required for each individual type as well as for potential mixtures [33]. In this regard, the composition of grape pomace can vary considerably depending on the crop variety and the processing techniques employed. Therefore, the chemical composition of cv. ‘Nero di Troia’ grape pomace was determined, before it was used as a mushroom growth substrate, and compared to wheat straw, as reported in Table 1.

**Table 1:** Chemical composition of wheat straw and grape pomace ^a^.

		Wheat Straw	Grape Pomace
pH		5.67 ± 0.01	3.93 ± 0.02
Moisture (105 °C)	%	6.2 ± 0.5	9.89 ± 0.10
Ash (550 °C)	%	5.0 ± 0.5	6.4 ± 0.6
TOC 400	%	40.2 ± 0.5	40.6 ± 0.2
ROC	%	0.20 ± 0.01	0.72 ± 0.02
TIC	%	0.19 ± 0.01	0.8 ± 0.1
TOC	%	40.4 ± 0.5	41.3 ± 0.3
TC	%	40.6 ± 0.5	42.1 ± 0.6
N	%	0.43 ± 0.02	1.25 ± 0.08
C/N		95 ± 8	33.6 ± 2.7
C_org_/N		94 ± 8	33.0 ± 2.4
CF	%	46.3 ± 0.5	34.5 ± 1.4
NDF	%	76.5 ± 1.8	57.9 ± 0.7
ADF	%	46.6 ± 1.3	53.0 ± 0.6
ADL	%	18.5 ± 1.6	16.6 ± 0.8

Results are expressed as the mean ± S.D. from 3 independent experiments. Abbreviations: TOC 400, total organic carbon oxidized at 400 °C; ROC, residual oxidizable carbon; TIC, total inorganic carbon; TOC, total organic carbon; TC, total carbon; N, nitrogen; C/N, carbon/nitrogen; C_org_/N, organic carbon/nitrogen; CF, crude fiber; NDF, neutral detergent fiber; ADF, acid detergent fiber; ADL, acid detergent lignin. ^a^ The results refer to dry material.

Grape pomace exhibited a markedly lower pH (3.93 ± 0.02), roughly similar to the value reported by Rodrigues et al. [34], compared to wheat straw (5.67 ± 0.01). The development of many fungal species depends on pH conditions; for instance, PO mycelium grows best in environments with a pH between 5 and 6.5 [35]. Moisture content was higher in grape pomace (9.89 ± 0.10%) than in wheat straw (6.2 ± 0.5%). Low humidity can cause the death of fruiting bodies. The ideal amount of moisture for growth and efficient use of the substrate varies depending on both the fungal species and the type of substrate employed. However, when moisture levels become too high, the substrate’s porosity decreases, which in turn restricts oxygen availability [36].

Ash concentration was also greater in grape pomace (6.4 ± 0.6%) as found in the values of other varieties analyzed [37], than in wheat straw (5.0 ± 0.5%). Both substrates showed comparable levels of total organic carbon (TOC), with values of 40.4 ± 0.5% for wheat straw and 41.3 ± 0.3% for grape pomace. The total carbon (TC) was found to be slightly higher in grape pomace (42.1 ± 0.6) compared to wheat straw (40.6 ± 0.5), according to what was previously reported [38]. TN content was almost three times higher in grape pomace (1.25 ± 0.08%) than in wheat straw (0.43 ± 0.02%), resulting in a narrower C/N ratio (33.6 ± 2.7 in grape pomace versus 95 ± 8 in wheat straw). An appropriate C/N ratio plays a central role in supporting healthy fungal growth and maximizing production [39]. Substrates with elevated nitrogen levels and consequently lower C/N ratios tend to promote stronger growth and improved yield performance [40]. If the nitrogen level exceeds the available carbon, the mushroom’s mycelium experiences excessive growth suppression [36]. A C/N ratio in the range of 22–30:1 is considered optimal for initiating primordia, with higher values favoring mycelial growth and lower values enhancing the development of fruiting bodies [41].

Regarding fiber fractions, wheat straw contained higher CF (46.3 ± 0.5%) and NDF (76.5 ± 1.8%) compared to grape pomace (34.5 ± 1.4% and 57.9 ± 0.7%, respectively). Conversely, ADF was higher in grape pomace (53.0 ± 0.6%) than in wheat straw (46.6 ± 1.3%). ADL values were comparable between the two materials (18.5 ± 1.6% in wheat straw and 16.6 ± 0.8% in grape pomace).

Table 2 illustrates the elemental composition and heavy metal content of wheat straw and grape pomace, expressed on a dry matter basis. The two substrates exhibited marked differences in their macro- and micronutrient profiles. Grape pomace contained substantially higher concentrations of potassium (31.8 ± 1.5 g kg^−1^ vs. 3.2 ± 0.8 g kg^−1^), calcium (6.8 ± 0.5 g kg^−1^ vs. 4.1 ± 0.2 g kg^−1^), and phosphorus (2162 ± 22 mg kg^−1^ vs. 220 ± 6 mg kg^−1^) compared to wheat straw. This condition can be advantageous, since the proper availability of both macro- and micronutrients is fundamental for fungal establishment and development. Phosphorus is particularly important, as it supports the synthesis of nucleic acids, membrane phospholipids, and ATP. In addition, elements such as potassium, magnesium, and trace minerals (including Ca, Mn, Fe, Zn, Cu, Ni, Co, and Mo) serve as enzyme cofactors and are indispensable for various metabolic processes [42].

**Table 2:** Elemental composition and heavy metal content of wheat straw and grape pomace.

		Wheat Straw	Grape Pomace
K _tot_	g kg^−1^	3.25 ± 0.8	31.8 ± 1.5
Ca _tot_	g kg^−1^	4.1 ± 0.2	6.8 ± 0.5
P _tot_	mg kg^−1^	220 ± 6	2162 ± 22
Mg _tot_	mg kg^−1^	507 ± 6	795 ± 9
Na _tot_	mg kg^−1^	690 ± 10	51.0 ± 2.6
Hg _tot_	mg kg^−1^	<0.5	<0.5
As _tot_	mg kg^−1^	<0.5	<0.5
Mo _tot_	mg kg^−1^	<0.5	<0.5
Zn _tot_	mg kg^−1^	26.2 ± 0.8	16.4 ± 0.3
Sb _tot_	mg kg^−1^	<0.5	<0.5
B _tot_	mg kg^−1^	11.7 ± 0.2	33.8 ± 1.1
Pb _tot_	mg kg^−1^	<0.5	<0.5
Co _tot_	mg kg^−1^	<0.5	<0.5
Cd _tot_	mg kg^−1^	<0.5	<0.5
Ni _tot_	mg kg^−1^	0.68 ± 0.05	<0.5
Fe _tot_	mg kg^−1^	168.0 ± 1.5	121.0 ± 1.9
Mn _tot_	mg kg^−1^	14.5 ± 0.1	14.6 ± 0.2
Cr _tot_	mg kg^−1^	0.88 ± 0.02	<0.5
V _tot_	mg kg^−1^	0.48 ± 0.09	<0.5
Cu _tot_	mg kg^−1^	6.4 ± 0.1	42.3 ± 0.5
Al _tot_	mg kg^−1^	252.0 ± 2.1	85.0 ± 1.8

Results are expressed as the mean ± S.D. from 3 independent experiments.

Magnesium content was also elevated in grape pomace (795 ± 9 mg kg^−1^) relative to wheat straw (507 ± 6 mg kg^−1^). Conversely, sodium levels were considerably higher in wheat straw (690 ± 10 mg kg^−1^) than in grape pomace (51.0 ± 2.6 mg kg^−1^). Regarding trace elements, grape pomace exhibited higher concentrations of boron (33.8 ± 1.1 mg kg^−1^ vs. 11.7 ± 0.2 mg kg^−1^) and copper (42.3 ± 0.5 mg kg^−1^ vs. 6.4 ± 0.1 mg kg^−1^), whereas zinc and iron were more abundant in wheat straw (26.2 ± 0.8 and 168.0 ± 1.5 mg kg^−1^, respectively) compared to grape pomace (16.4 ± 0.3 and 121.0 ± 1.9 mg kg^−1^, respectively). Manganese concentrations were comparable between the two matrices (14.5 ± 0.1 mg kg^−1^ in wheat straw vs. 14.6 ± 0.2 mg kg^−1^ in grape pomace).

With respect to heavy metals, both substrates showed values below the detection limit (<0.5 mg kg^−1^) for mercury, arsenic, molybdenum, antimony, lead, cobalt, cadmium, nickel (in grape pomace), chromium (in grape pomace), and vanadium (in grape pomace). However, wheat straw contained detectable but low concentrations of nickel (0.68 ± 0.05 mg kg^−1^), chromium (0.88 ± 0.02 mg kg^−1^), and vanadium (0.48 ± 0.09 mg kg^−1^), which were absent in grape pomace. Aluminum was more abundant in wheat straw (252.0 ± 2.1 mg kg^−1^) than in grape pomace (85.0 ± 1.8 mg kg^−1^). Overall, grape pomace demonstrated a richer profile in essential macronutrients (K, Ca, P, Mg) and certain micronutrients (B, Cu), while wheat straw was characterized by higher levels of Na, Zn, Fe, Al, and trace amounts of some heavy metals. These compositional differences may influence their potential applications in fugal cultivation, and it is quite common for various mushroom species to show distinct preferences in the way they take up mineral elements [43].

### 3.2. Mushroom Microwave-Assisted Extraction

The worldwide growth of mushroom production is sustained by their richness in nutrients and their unique importance in gastronomy [44]. Moreover, inadequate handling of agro-industrial residues and the open burning of agricultural by-products negatively affect the environment and pose risks to human health. Since they are cultivated on lignocellulosic materials, mushroom cultivation effectively transforms low-value biomass into high-quality food [45]. Fungi, especially species belonging to the genus *Pleurotus*, represent valuable agents for advancing CE strategies, as they enable sustainable waste valorization, biotransformation processes, and contribute to environmental restoration [46]. Mushroom cultivation represents a clear example of CE principles, as it converts organic residues into valuable biomass by decomposing and recycling nutrients from organic waste [47].

In the framework of a CE, the effective handling and valorization of winery residues and by-products play a key role in advancing sustainable development. In recent years, grape pomace has gained growing attention in the food sector thanks to its rich composition of nutritional and bioactive constituents such as polyphenols, proanthocyanidins, flavonoids, and phenolic acids [48]. It has been demonstrated that grape pomace is a valuable waste product for mushroom cultivation, both from the point of view of environmental sustainability and the quality of the mushroom extract cultivated on this waste product [49]. Despite being considered a residue, the grape pomace retains significant amounts of health-promoting compounds, highlighting its importance as a sustainable and precious resource [50]. In fact, the winemaking by-product is rich in phenolic compounds such as anthocyanins, flavonoids, and stilbenes, thus representing a valuable source of bioactive molecules that influence different aspects of human biology and offer potential benefits for applications in the agri-food sector [51]. In particular, we focused our attention on grape pomace from the cv. ‘Nero di Troia’, never explored so far as growth substrate for mushrooms.

Both fungal species *P. ostreatus* (PO) and *P. eryngii* var. *eryngii* (PE) were cultivated on three substrate types: wheat straw (S), grape pomace (P), and an equal dry-weight mixture (M) of wheat straw and grape pomace. For PO, the analyzed samples were designated as POS, POP, and POM, while for PE, the corresponding samples were labeled PES, PEP, and PEM.

MAE technique of bioactive compounds from different natural matrices [16,52,53], including mushrooms [26,54] was intensively investigared in our previous works. MAE was selected since it represents a sustainable and innovative approach that offers clear benefits compared to traditional extraction methods, including faster extraction kinetics, shorter processing times, reduced use of solvents, and decreased energy requirements [55]. These green extraction methods have become a game-changing strategy in biotechnology, especially for overcoming the difficulties of breaking down fungal cell walls to improve protein recovery and production [56].

### 3.3. Antioxidant Activity Evaluation

**Figure 1:**
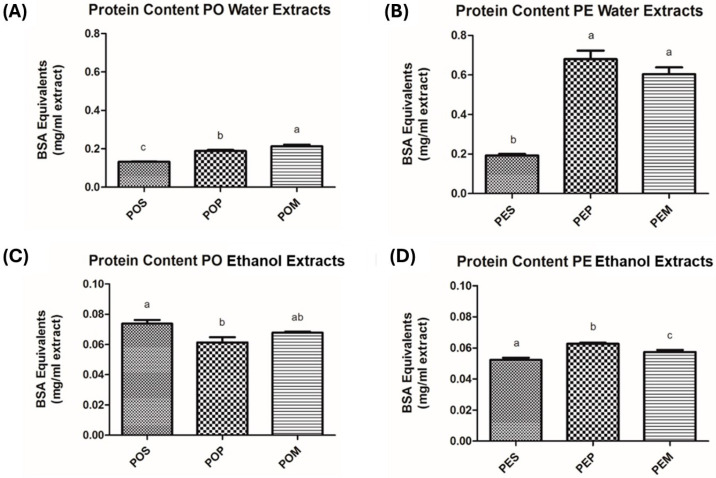
Protein content of the six prepared mushroom extracts expressed as BSA Equivalents (mg/mL extract): (**A**) Protein Content of *P. ostreatus* Water Extracts; (**B**) Protein Content of *P. eryngii* var. *eryngii* Water Extracts; (**C**) Protein Content of *P. ostreatus* Ethanol Extracts; (**D**) Protein Content of *P. eryngii* var. *eryngii* Ethanol Extracts. The results are expressed as means ± S.D. from at least three independent experiments. *p*-Values were determined using a one-way ANOVA followed by Tukey–Kramer post hoc test. Values with the same letter are not significantly different at α = 0.05. Abbreviations: PO, *Pleurotus ostreatus*; PE, *Pleurotus eryngii* var. *eryngii*; POS, *Pleurotus ostreatus* wheat straw; POP, *Pleurotus ostreatus* grape pomace; POM, *Pleurotus ostreatus* mixture; PES, *Pleurotus eryngii* var. *eryngii* wheat straw; PEP, *Pleurotus eryngii* var. *eryngii* grape pomace; PEM, *Pleurotus eryngii* var. *eryngii* mixture; BSA, bovine serum albumin.

**Figure 2:**
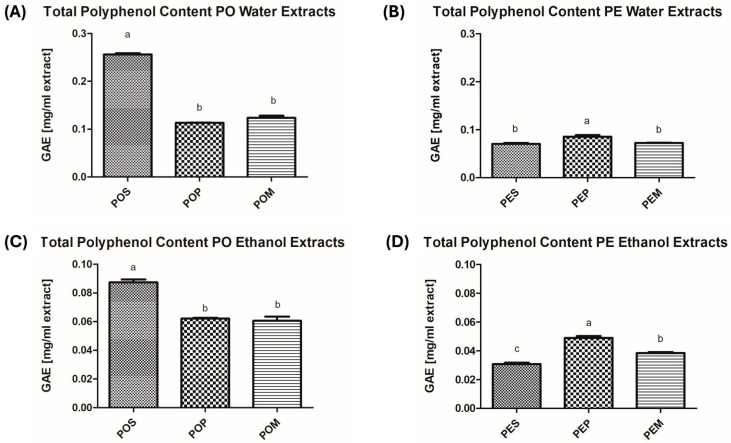
Polyphenol content of the six prepared mushroom extracts expressed as GA Equivalents (mg/mL extract). (**A**) Total Polyphenol Content of *P. ostreatus* Water Extracts; (**B**) Total Polyphenol Content of *P. eryngii* var. *eryngii* Water Extracts; (**C**) Total Polyphenol Content of *P. ostreatus* Ethanol Extracts; (**D**) Total Polyphenol Content of *P. eryngii* var. *eryngii* Ethanol Extracts. The results are expressed as means ± S.D. from at least three independent experiments. *p*-Values were determined using a one-way ANOVA followed by Tukey–Kramer post hoc test. Values with the same letter are not significantly different at α = 0.05. Abbreviations: PO, *Pleurotus ostreatus*; PE, *Pleurotus eryngii* var. *eryngii*; POS, *Pleurotus ostreatus* wheat straw; POP, *Pleurotus ostreatus* grape pomace; POM, *Pleurotus ostreatus* mixture; PES, *Pleurotus eryngii* var. *eryngii* wheat straw; PEP, *Pleurotus eryngii* var. *eryngii* grape pomace; PEM, *Pleurotus eryngii* var. *eryngii* mixture; GAE, gallic acid equivalents.

**Figure 3:**
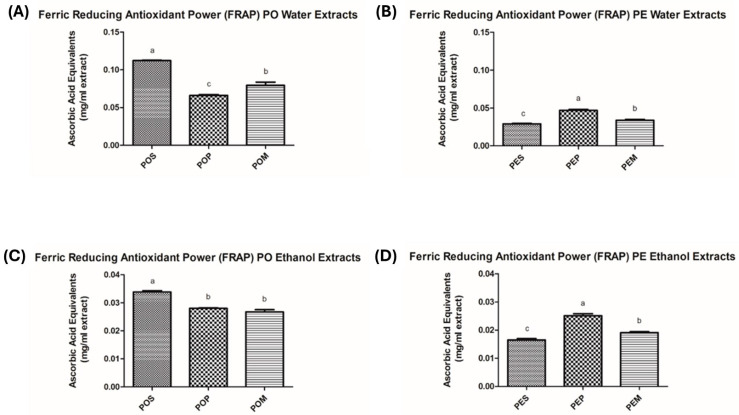
Effects of different mushroom extracts on Ferric Reducing Antioxidant Power (FRAP) expressed as AA Equivalents (mg/mL extract). (**A**) Ferric Reducing Antioxidant Power (FRAP) of *P. ostreatus* Water Extracts; (**B**) Ferric Reducing Antioxidant Power (FRAP) of *P. eryngii* var. *eryngii* Water Extracts; (**C**) Ferric Reducing Antioxidant Power (FRAP) of *P. ostreatus* Ethanol Extracts; (**D**) Ferric Reducing Antioxidant Power (FRAP) of *P. eryngii* var. *eryngii* Ethanol Extracts. The results are expressed as means ± S.D. from at least three independent experiments. *p*-Values were determined using a one-way ANOVA followed by Tukey–Kramer post hoc test. Values with the same letter within columns are not significantly different at α = 0.05. Abbreviations: PO, *Pleurotus ostreatus*; PE, *Pleurotus eryngii* var. *eryngii*; POS, *Pleurotus ostreatus* wheat straw; POP, *Pleurotus ostreatus* grape pomace; POM, *Pleurotus ostreatus* mixture; PES, *Pleurotus eryngii* var. *eryngii* wheat straw; PEP, *Pleurotus eryngii* var. *eryngii* grape pomace; PEM, *Pleurotus eryngii* var. *eryngii* mixture.

**Figure 4:**
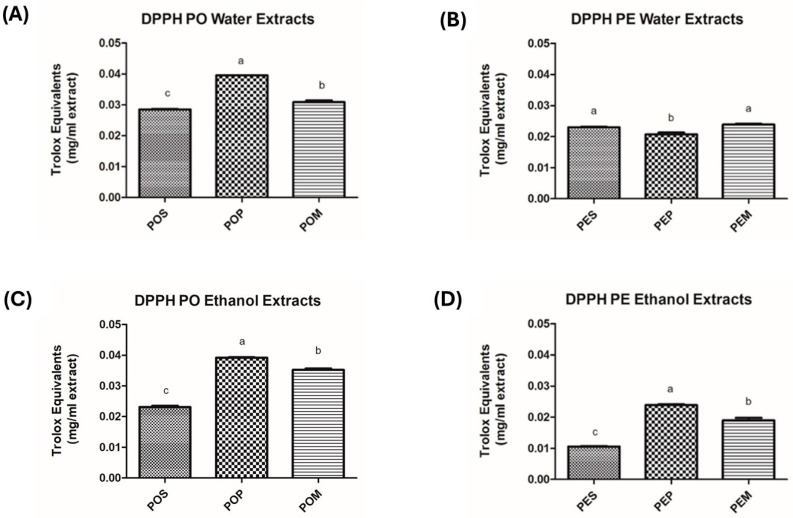
Effects of different mushroom extracts on radical scavenging activity (DPPH) expressed as TROLOX equivalents (mg/mL extract). (**A**) DPPH of *P. ostreatus* Water Extracts; (**B**) DPPH of *P. eryngii* var. *eryngii* Water Extracts; (**C**) DPPH of *P. ostreatus* Ethanol Extracts; (**D**) DPPH of *P. eryngii* var. *eryngii* Ethanol Extracts. The results are expressed as means ± S.D. from at least three independent experiments. *p*-Values were determined using a one-way ANOVA followed by Tukey–Kramer post hoc test. Values with the same letter within columns are not significantly different at α = 0.05. Abbreviations: PO, *Pleurotus ostreatus*; PE, *Pleurotus eryngii* var. *eryngii*; POS, *Pleurotus ostreatus* wheat straw; POP, *Pleurotus ostreatus* grape pomace; POM, *Pleurotus ostreatus* mixture; PES, *Pleurotus eryngii* var. *eryngii* wheat straw; PEP, *Pleurotus eryngii* var. *eryngii* grape pomace; PEM, *Pleurotus eryngii* var. *eryngii* mixture; DPPH, 1,1-diphenyl-2-picrylhydrazyl.

Starting from the consideration that polyphenols are endowed with antioxidant activity and are generally soluble in polar solvents [57], all available *Pleurotus* samples (POS, POP, POM, PES, PEP, PEM) underwent extraction with water or absolute EtOH to conceivably evaluate a different biological profile among the extracts in terms of phytochemical content and, accordingly, biological activity. In particular, the total protein content (expressed as mg of bovine serum albumin equivalents (BSAeq.)/mL extract), total polyphenol content (TPC, expressed as mg of gallic acid equivalents (GAE)/mL extract), and antioxidant activity (evaluated through the FRAP and DPPH assays) were assessed. The results are reported in Figure 1, Figure 2, Figure 3 and Figure 4 and Table A1, Table A2, Table A3 and Table A4.

In general, a 2–10-fold higher extract protein content was observed for both mushroom species when extraction was carried out with water (Figure 1A,B, Table A1) compared to the extraction with EtOH (Figure 1C,D, Table A1). As regards the water extracts (Figure 1A,B, Table A1), PE gave higher protein content than PO, regardless of the growth substrate, the difference being higher when the mushrooms were cultivated exclusively or partially on grape pomace (PEP and PEM, respectively). The extract content should reflect the corresponding mushroom protein content, thus underlining the usefulness of the grape pomace waste as a PE growth substrate to obtain a high-value-added food. High protein intakes help prevent and/or treat obesity, maximize athletic performance, and may help prevent age-related sarcopenia [58].

As observed for protein content, a higher polyphenol content was found when water was used as the extraction solvent (Figure 2A,B, Table A2) compared to EtOH (Figure 2C,D, Table A2), with PO always showing higher content values than PE. Focusing our attention on the results related to grape pomace (POP and PEP), the water extract from PO showed a TPC value of 0.113 ± 0.002 mg GAE/mL (POPW) compared to 0.085 ± 0.008 mg GAE/mL obtained for PEPW.

A similar trend was observed for the antioxidant activity, in terms of ferric reducing antioxidant power (FRAP) (Figure 3 and Table A3): water proved to be a more effective extraction solvent, and PO showed a higher antioxidant power than PE. In particular, when grape pomace was used as substrate, an ascorbic acid equivalent (AAE) value of 0.066 ± 0.004 mg/mL was obtained for the water extract of POPW compared to 0.046 ± 0.003 mg/mL for PEPW.

A different extract’s behavior was observed only for the DPPH assay, where only mild differences were registered between the two extraction solvents for both mushroom species. Anyway, regardless of the extraction solvent used, the more interesting results were obtained for PO growth on grape pomace (Figure 4 and Table A4), with 0.040 ± 0.001 TROLOX equivalents/mL obtained for water extract (POPW) and 0.039 ± 0.001 TROLOX equivalents/mL obtained for alcoholic extract (POPE) being the highest values in the series.

Interestingly, although both capable of assessing antioxidant activity, FRAP and DPPH assays provide different information and are related to different classes of compounds. The antioxidant activity revealed through the FRAP assay is mostly related to hydrophilic compounds that are endowed with reducing power stemming from electron-donating properties. The antioxidant activity displayed in the DPPH assay is related to both hydrophilic and hydrophobic free radical scavengers. Therefore, based on the results obtained, grape pomace is a valuable waste material that can be used as a growth medium for cultivating mushrooms, possibly endowed with antioxidant properties in terms of radical scavenging activity. In general, the mixture of wheat straw and grape pomace (POM and PEM) yielded intermediate results compared to the individual components, with unnoteworthy exceptions.

### 3.4. GC-MS Profiling of P. ostreatus and P. eryngii Extracts

The extracts obtained with both solvents from PO and PE grown on grape pomace (POPW, POPE, PEPW, PEPE) were first analyzed by GC-MS to investigate the potential effect of such cultivation on the chemical composition of the resulting mushrooms. The identification of volatile metabolites was carried out by matching the mass spectral fragmentation patterns of each revealed analyte with those of known compounds in the NIST library or previously described in the literature. The identified metabolites, along with their retention times, relative peak areas (%), key ion species, and corresponding chemical classes, are listed in Table 3 and Table 4. All the compounds are arranged in order of their elution progression.

Based on the peak area evaluation, the most abundant phytochemical recorded in the POP and PEP ethanol extracts (Table 3 and Table 4, respectively) is sorbitol, a polyol well known for its laxative properties in treating constipation and also endowed with mild antioxidant properties [59]. Therefore, mushrooms rich in sorbitol might help relieve constipation and related ROS-sustained inflammatory states. Starting from the consideration that *Pleurotus* mushrooms do not naturally contain sorbitol, it is possible to assume that mushrooms cultivated on grape pomace soak up this sugar alcohol from the growth medium. For the sake of truth, the highest level of sorbitol is contained in grape pulp rather than grape pomace since it is synthesized in leaves and transported into the berry pulp [60]. Considering that the skins, abundantly contained in grape pomace, are in contact with the pulp and phloem tissues, and that grape pomace often retains some adhering pulp or juice, the presence of residual sorbitol can be hypothesized in grape pomace. To confirm our hypothesis, the PESE extract was also analyzed by GC-MS, and sorbitol was actually not detected.

Palmitic acid (PA) and linoleic acid (LA) were detected in the POP alcoholic extract, with the saturated fatty acid LA content being about 20-fold higher than the polyunsaturated fatty acid PA. Besides PA and LA, oleic acid (OA) was also detected in the PEP alcoholic extract, in a percentage similar to that of LA. The PEP total content of unsaturated fatty acids was about 18-fold higher than that of PA. Considering that PA is involved in the beta-amyloid peptide (Aβ) production, which plays a pivotal role in AD pathogenesis [61,62], its low content with respect to unsaturated fatty acids in both extracts highlights grape pomace as a waste material valuable as a growth medium in mushroom cultivation. As regards the unsaturated fatty acids, PE showed a more interesting chemical composition: not only was its LA percentage about 3-fold higher than that observed in PO, but OA was also detected. LA has been associated with anti-inflammatory, hypocholesterolemic, and anticancer properties [63]. OA is known for its anti-inflammatory, neuroprotective, and cardioprotective activities [64]. Summing up these findings, cultivation of PE on grape pomace should be particularly recommended. Ergosterol and the glycerol monoester of both palmitic and stearic acids were also recorded in both extracts, in percentages lower than those of unsaturated fatty acids.

Unfortunately, results relating to POP and PEP water extracts chemical profiles are missing because no data were collected from the detector (only background noise was recorded), probably because of low solubility and/or instability of the secondary metabolites under the analytical experimental conditions (GC-MS analysis). Conceivably, the highly polar bioactive compounds extracted with water (sugars first) did not dissolve in methanol used for injection, with methanol being the most polar organic solvent allowed in GC-MS. In fact, although methanol is a polar protic solvent, it has a lower hydrogen-bonding capacity compared to water, so that the solubility of some sugars is very low compared to water.

It is known that grape pomace seeds are rich in lipids, which are not soluble in the two previously adopted solvents (water and EtOH). Therefore, to recover a higher amount of lipophilic phytochemicals possibly absorbed by our mushrooms from grape pomace, a green solvent with a lower polarity degree than water and EtOH was chosen, namely ethyl acetate [57]. All the obtained extracts (POSA, POPA, POMA, PESA, PEPA, PEMA) were evaluated for their chemical composition through GC-MS analysis, as reported in Table 5 and Table 6.

In general, all six extracts contained fatty acids—namely, palmitic, linoleic, and oleic acids (PA, LA, and OA, respectively)—and ergosterol. No substantial difference in PA content related to the used substrate was observed for the PO species, unlike what was observed for PE, whose PA content was about 8-fold higher when mushroom cultivation was carried out on wheat straw rather than on grape pomace. Given the known role of PA in neurodegenerative diseases, due to its involvement in the Aβ production, the low PA percentage (<1%) found in the PEPA extract promotes the use of grape pomace as a more valuable growth medium for PE than wheat straw [61,62].

As reported above for hydrophilic extracts, PE also showed higher levels of OA compared to PO, regardless of the growth medium. On the contrary, LA was more abundant in the extract of the mushroom grown on grape pomace compared to wheat straw for both mushroom species (compare POPA 53% and PEPA 49% with POSA 18% and PESA 19%). On the other hand, it is well known that grape seeds of grapevine varieties are rich in fatty acids, with LA usually being the most abundant [65]. The LA content found for PE grown on a mixture of wheat straw-grape pomace (50%) confirms the above considerations. With similar LA values for the two mushroom species, PE should be preferred due to the abundance of the healthy OA in its extracts [64].

As regards sterols, the highest relative abundance of ergosterol was registered for the POPA extract, immediately followed by PEPA. Starting from the consideration that ergosterol’s antioxidant properties have been reported [66], our results confirm the usefulness of wine waste materials to obtain high-value-added foods.

Interestingly, fatty alcohols (1-tetradecanol, 1-pentadecanol, 1-hexadecanol, 1-heptadecanol, 1-nonadecanol, 1-heneicosanol, behenic alcohol, and 1-tetracosanol) were tentatively identified only in extracts obtained from PO. The presence of several fatty alcohols in mushrooms has already been described in the literature [67]. In general, fatty alcohols are known to be endowed with antimicrobial properties [68]. Together with the two monoacylglycerols detected, fatty alcohols display emollient or emulsifying properties [69,70]. Both antimicrobial and tensioactive properties should stem from their amphiphilic nature and resulting ability to interact with membranes and lipids. Therefore, in-depth evaluation of PO extracts in the food industry as emulsifiers or in cosmetics as emollients and skin conditioners, possibly displaying conservative properties, will be carried out in the future.

2,4-Di-tert-butylphenol [phenol, 2,4-bis(1,1-dimethylethyl)] was also detected in both PO extracts (POS and POP). This antioxidant, anti-inflammatory, antimicrobial, and anticancer phenol has already been detected in edible mushrooms, according to what was previously reported in the literature [71].

Unfortunately, the POMA extract exhibited peculiar behavior in the aqueous solution before GC-MS injection, as a precipitate formed. To inject only the soluble fraction, the precipitate was filtered off, which likely resulted in the loss of some analytes. Furthermore, some difficulties were encountered in recording the results (perhaps due to the resulting excessive dilution and thermal degradation of the sample), and this is why only a few substances were detected, with their relative areas not determined (see Table 5).

### 3.5. Acetylcholinesterase Inhibitory Activity Determination

It has recently been reported that LA showed inhibitory activity on acetylcholinesterase (AChE) [72], a well-known serine-hydrolase enzyme responsible for the catabolic degradation of the neurotransmitter acetylcholine (ACh). The blockade of AChE activity in different organs is the cornerstone for treating different human diseases, particularly within the central nervous system, as a tool against Alzheimer’s disease. AD represents the main cause of senile dementia, with a growing impact on the elderly population. This neurodegenerative pathology is associated with the progressive decline of spatiotemporal and cognitive functions involving brain regions under cholinergic control. Thus, the restoration of adequate levels of the neurotransmitter has long been considered a viable strategy to counterbalance ACh depletion. Even if AChE inhibition exerts only symptomatic effects, this enzymatic target still attracts the attention of researchers working on anti-AD multitarget or polypharmacology approaches [73]. As a matter of fact, one of the most recently approved drug (trade name Namzaric) is a combination of two active ingredients, e.g., memantine (an NMDA-antagonist) and donepezil (a reversible AChE inhibitor). On the other hand, there is a growing interest in plant-derived AChE inhibitors, among which galantamine is one of the few marketed natural drugs approved for symptomatic treatment in mild to moderate AD patients [74]. Interesting results have also been reported for berberine [75] whose effects on cognitive function and β-amyloid precursor protein in AD models have been reviewed [76].

To the best of our knowledge, no studies on *Pleurotus* ethyl acetate extracts obtained under microwave irradiation have ever been conducted so far. Therefore, both POPA and PEPA, the most interesting extracts in terms of CE, also showing high levels of LA, were tested in vitro for their ability to inhibit the enzymatic activity of human acetylcholinesterase (hAChE) by applying the spectrophotometric Ellmann’s method, as already reported [32]. As displayed in Table 7, both POPA and PEPA returned promising in vitro inhibitory potencies at all tested concentrations compared to donepezil as a standard anti-AChE marketed drug, deserving further attention in this context.

## 4. Conclusions

The present study sheds light on grape pomace from cv. ‘Nero di Troia’, a winemaking by-product, as a promising alternative substrate to conventional wheat straw for the cultivation of *Pleurotus* species. From a CE perspective, valorizing winery residues through mushroom cultivation exemplifies an environmentally friendly strategy for reducing agricultural waste, decreasing open-field burning practices, and generating value-added products with potential applications in the agri-food and nutraceutical sectors. We demonstrated that the use of grape pomace, either alone or in combination with wheat straw, not only supports fungal growth but also enhances the nutritional and functional quality of the mushroom extracts obtained under microwave irradiation. The usefulness of the eco-friendly microwave-assisted extraction technique as an efficient and sustainable method to recover bioactive compounds was thus confirmed. The more interesting biological results were observed for both mushroom species (*P. ostreatus* and *P. eryngii* var. *eryngii*) cultivated on cv. ‘Nero di Troia’ grape pomace and extracted under microwave irradiation using ethyl acetate as the extraction solvent. The corresponding extracts showed promising in-vitro inhibitory activity against AChE, an enzyme involved in the pathogenesis of AD. Overall, the results highlight that integrating grape pomace into mushroom production systems represents a viable step toward more sustainable and resource-efficient biotechnological processes. This approach contributes to the development of circular bioeconomy models that combine waste valorization, sustainable food production, and the generation of bioactive-rich products with potential benefits for human health. These interesting preliminary results encourage research to expand on and deepen the areas of application of the extracts, such as studies on the inhibition of tumor cell proliferation and antimicrobial and antibiofilm assays. The technique of adding grape pomace to the substrate could be applied to the cultivation of other medicinal mushroom species to improve their bioactive activities.

## Figures and Tables

**Table 3:** Bioactive compounds tentatively identified by GC-MS analysis in POP EtOH extract (POPE).

Compound	Molecular Formula	*M*_r_ (Nominal; g/mol)	RT ^a^ (min)	Peak Area (%)	Key Ion Species *m*/*z*	Chemical Class
*n*-Hexadecanoic acid (Palmitic acid)	C_16_H_32_O_2_	256	40.28	0.3	73 (100), 256 (M^+^, 2.3)	Saturated Carboxylic Acids
9,12-Octadecadienoic acid (Linoleic acid, LA)	C_18_H_32_O_2_	280	44.14	5.8	67 (100), 280 (M^+^, 7.6)	Fatty acid (polyunsaturated, essential)
Sorbitol	C_6_H_14_O_6_	182	50.82	86.3	73 (100), 133 (M^+^ −49, 9.1)	Polyols
Glycerol monopalmitate	C_19_H_38_O_4_	330	51.76	4.1	43 (100), 299 (M^+^ −31, 3.5)	Esters (glycerides)
Glycerol monostearate	C_21_H_42_O_4_	358	55.57	1.8	98 (100), 298 (M^+^ −60, 3.3)	Esters (glycerides)
Ergosterol	C_28_H_44_O	396	63.39	1.7	55 (100), 396 (M^+^, 23.7)	Sterols

^a^ RT: retention time.

**Table 4:** Bioactive compounds tentatively identified by GC-MS analysis in PEP EtOH extract (PEPE).

Compound	Molecular Formula	*M*_r_ (Nominal; g/mol)	RT ^a^ (min)	Peak Area (%)	Key Ion Species *m*/*z*	Chemical Class
*n*-Hexadecanoic acid (Palmitic acid)	C_16_H_32_O_2_	256	40.22	1.7	73 (100), 256 (M^+^, 13.0)	Saturated Carboxylic Acids
9,12-Octadecadienoic acid (Linoleic acid, LA)	C_18_H_32_O_2_	280	44.14	16.7 ^b^	67 (100), 280 (M^+^, 4.8)	Fatty acid (polyunsaturated, essential)
9-Octadecenoic acid (Oleic acid, OA)	C_18_H_34_O_2_	282	44.26	14.7 ^b^	55 (100), 282 (M^+^, <1)	Fatty acid (monounsaturate, essential)
Sorbitol	C_6_H_14_O_6_	182	47.50	62.9	73 (100), 133 (M^+^ −49, 9.0)	Polyols
Glycerol monopalmitate	C_19_H_38_O_4_	330	51.80	0.9	43 (100), 239 (M^+^ −91, 33.3)	Esters (glycerides)
Glycerol monostearate	C_21_H_42_O_4_	358	55.58	0.9	98 (100), 267 (M^+^ −91, 19.3)	Esters (glycerides)
Ergosterol	C_28_H_44_O	396	63.39	1.6	69 (100), 396 (M^+^, 17.5)	Sterols

^a^ RT: retention time. ^b^ Rough peak area values relating to two chromatogram peaks not baseline resolved.

**Table 5:** Bioactive compounds identified by GC-MS analysis in PO ethyl acetate extracts.

			POSA			POPA			POMA			
Compound	Molecular Formula	*M*_r_ (Nominal; g/mol)	RT ^a^ (min)	Peak Area (%)	Key Ion Species *m*/*z*	RT ^a^ (min)	Peak Area (%)	Key Ion Species *m*/*z*	RT ^a^ (min)	Peak Area (%)	Key Ion Species *m*/*z*	Chemical Class
1-Tetradecanol	C_14_H_30_O	214	25.08	2.38	55 (100), 196 (M^+^ −18, 2.5)	25.10	0.59	55 (100), 196 (M^+^ −18, 2.5)	n.d. ^a^			Fatty alcohols
Phenol, 2,4-bis(1,1-dimethylethyl)	C_14_H_22_O	206	28.82	7.83	191 (100), 206 (M^+^, 15.6)	28.86	0.32	191 (100), 206 (M^+^, 13.2)	n.d. ^a^			Phenols
1-Pentadecanol	C_15_H_32_O	228	31.11	3.11	55 (100), 140 (M^+^ −88, 3.7)	31.12	1.06	55 (100), 140 (M^+^ −88, 2.6)	n.d. ^a^			Fatty alcohols
1-Hexadecanol	C_16_H_34_O	242	36.55	2.23	55 (100), 140 (M^+^ −102, 5.1)	36.55	0.86	55 (100), 140 (M^+^ −102, 2.4)	n.d. ^a^			Fatty alcohols
1-Heptadecanol	C_17_H_36_O	256	38.77	2.01	55 (100), 140 (M^+^ −116, 4.0)	38.78	0.54	55 (100), 140 (M^+^ −116, 3.0)	n.d. ^a^			Fatty alcohols
*n*-Hexadecanoic acid (Palmitic acid)	C_16_H_32_O_2_	256	40.87	10.15	73 (100), 256 (M^+^, 14.4)	40.95	8.92	73 (100), 256 (M^+^, 15.1)	n.d. ^a^			Saturated Carboxylic Acids
1-Nonadecanol	C_19_H_40_O	284	41.48	1.32	83 (100), 154 (M^+^ −130, 4.4)	41.48	0.70	83 (100), 168 (M^+^ −116, 1.9)	n.d. ^a^			Fatty alcohols
1-Heneicosanol	C_21_H_44_O	312	43.57	2.31	83 (100), 153 (M^+^ −159, 15.3)	43.57	0.80	69 (100), 224 (M^+^ −88, 2.7)	n.d. ^a^			Fatty alcohols
9,12-Octadecadienoic acid (Linoleic acid, LA)	C_18_H_32_O_2_	280	44.81	18.42 ^b^	67 (100), 280 (M^+^, 7.7)	45.06	53.24 ^b^	67 (100), 280 (M^+^, 9.4)	44.62	n.d.	67 (100), 280 (M^+^, 3.1)	Fatty acid (polyunsaturated, essential)
9-Octadecenoic acid (Oleic acid, OA)	C_18_H_34_O_2_	282	44.90	6.72 ^b^	55 (100), 264 (M^+^ −18, 6.5)	45.11	6.97 ^b^	55 (100), 282 (M^+^, <1)	n.d. ^a^			Fatty acid (monounsaturated, essential)
Behenic alcohol	C_22_H_46_O	326	45.99	0.58	55 (100), 125 (M^+^ −201, 9.0)	45.99	0.29	55 (100), 280 (M^+^ −46, 2.8)	n.d. ^a^			Fatty alcohols
Glycerol monopalmitate	C_19_H_38_O_4_	330	52.42	6.99	43 (100), 299 (M^+^ −31, 5.7)	52.43	1.34	43 (100), 270 (M^+^ −60, 2.6)	n.d. ^a^			Esters (glycerides)
Glycerol monostearate	C_21_H_42_O_4_	358	56.25	8.33	43 (100), 327 (M^+^ −31, 2.9)	56.25	1.14	43 (100), 327 (M^+^ −31, 2.4)	n.d. ^a^			Esters (glycerides)
1-Tetracosanol	C_24_H_50_O	354	62.63	1.03	57 (100), 139 (M^+^ −215, 11.7)	62.65	0.27	57 (100), 139 (M^+^ −215, 37.3)	n.d. ^a^			Fatty alcohols
Ergosterol	C_28_H_44_O	396	64.17	3.64	69 (100), 396 (M^+^, 37.3)	64.21	14.90	69 (100), 396 (M^+^, 41.5)	63.88	n.d.	69 (100), 396 (M^+^, 16.7)	Sterols

^a^ RT: retention time. ^b^ Rough peak area values relating to two chromatogram peaks (at 45.06 and 45.11 min, respectively) not baseline resolved. n.d.: not determined.

**Table 6:** Bioactive compounds identified by GC-MS analysis in PE ethyl acetate extracts.

			PESA			PEPA			PEMA			
Compound	Molecular Formula	*M*_r_ (Nominal; g/mol)	RT ^a^ (min)	Peak Area (%)	Key Ion Species *m*/*z*	RT ^a^ (min)	Peak Area (%)	Key Ion Species *m*/*z*	RT ^a^ (min)	Peak Area (%)	Key Ion Species *m*/*z*	Chemical Class
*n*-Hexadecanoic acid (Palmitic acid)	C_16_H_32_O_2_	256	40.60	0.91	73 (100), 256 (M^+^, 16.6)	40.62	7.67	73 (100), 256 (M^+^, 20.3)	40.59	1.14	73 (100), 256 (M^+^, 15.9)	Saturated fatty acids
9,12-Octadecadienoic acid (Linoleic acid, LA)	C_18_H_32_O_2_	280	44.59	48.81 ^b^	67 (100), 280 (M^+^, 13.3)	44.61	18.87 ^b^	67 (100), 280 (M^+^, 10.4)	44.61	50.43 ^b^	67 (100), 280 (M^+^, 11.5)	Fatty acid (polyunsaturated, essential)
9-Octadecenoic acid (Oleic acid, OA)	C_18_H_34_O_2_	282	44.70	38.19 ^b^	55 (100), 282 (M^+^, 1.3)	44.72	54.22 ^b^	55 (100), 282 (M^+^, 2.6)	44.70	36.72 ^b^	55 (100), 282 (M^+^, 2.1)	Fatty acid (monounsaturated, essential)
Ergosterol	C_28_H_44_O	396	63.82	7.39	69 (100), 396 (M^+^, 80.3)	63.85	2.41	69 (100), 396 (M^+^, 34.3)	63.83	6.12	69 (100), 396 (M^+^, 77.2)	Sterols

^a^ RT: retention time. ^b^ Rough peak area values relating to two chromatogram peaks (at 44.61 and 44.72 min, respectively) not baseline resolved.

**Table 7:** Evaluation of the POPA and PEPA extracts’ acetylcholinesterase inhibitory activity.

POPA		PEPA	
Concentration	*h*AChE % Inhibition	Concentration	*h*AChE % Inhibition
3.0 mg/mL	61 ± 6%	4.0 mg/mL	81 ± 5%
1.5 mg/mL	51 ± 5%	2.0 mg/mL	75 ± 4%
1.0 mg/mL	44 ± 3%	1.5 mg/mL	69 ± 4%
	***h*AChE (IC_50_, μM)**		
donepezil	0.0220 ± 0.0011		

Results are expressed as the mean ± S.D. from 3 independent experiments. Abbreviations: POPA, *Pleurotus ostreatus* grape pomace ethyl acetate; PEPA, *Pleurotus eryngii* var. *eryngii* grape pomace ethyl acetate.

## Data Availability

The data that support the findings of this study are available upon request to the corresponding author.

## Abbreviations

The following abbreviations are used in this manuscript:

A	Ethyl acetate
AAE	Ascorbic acid equivalent
ACh	Acetylcholine
AChE	Acetylcholinesterase
AD	Alzheimer’s disease
ADF	Acid detergent fiber
ADL	Acid detergent lignin
AMU	Atomic mass units
Aβ	Beta-amyloid peptide
BSA	Bovine serum albumin
CE	Circular economy
CF	Crude fiber
DPPH	1,1-diphenyl-2-picrylhydrazyl
E	Absolute ethanol
FRAP	Ferric reducing antioxidant power
GAE	Gallic acid equivalents
GC-MS	Gas chromatography-mass spectrometry
LA	Linoleic acid
M	Mixture
MAE	Microwave-assisted extraction
NIST	National Institute of Standards and Technology
NDF	Neutral detergent fiber
NMDA	N-methyl-D-aspartate
OA	Oleic acid
P	Grape pomace
PA	Palmitic acid
ROC	Residual oxidizable carbon
S	Wheat straw
S.D.	Standard deviation
TC	Total carbon
TIC	Total inorganic carbon
TN	Total nitrogen
TOC	Total organic carbon
TOC 400	Total organic carbon oxidized at 400 °C
TPC	Total polyphenol content
W	Water

## Appendix A

### Appendix A.1

**Table A1:** Total protein content of mushroom extracts.

	Bovine Serum Albumin Equivalents (mg/mL Extract)					
	POS	POP	POM	PES	PEP	PEM
water (W)	0.131 ± 0.004 ^c^	0.190 ± 0.018 ^b^	0.213 ± 0.026 ^a^	0.192 ± 0.025 ^b^	0.68 ± 0.14 ^a^	0.60 ± 0.11 ^a^
abs. EtOH (E)	0.074 ± 0.007 ^a^	0.061 ± 0.010 ^b^	0.068 ± 0.004 ^ab^	0.052 ± 0.004 ^c^	0.062 ± 0.001 ^a^	0.057 ± 0.003 ^b^

The results are expressed as means ± S.D. from at least three independent experiments. *p*-Values were determined using a one-way ANOVA followed by Tukey–Kramer post hoc test. Values with the same letter within a line are not significantly different at α = 0.05. Abbreviations: POS, *Pleurotus ostreatus* wheat straw; POP, *Pleurotus ostreatus* grape pomace; POM, *Pleurotus ostreatus* mixture; PES, *Pleurotus eryngii* var. *eryngii* wheat straw; PEP, *Pleurotus eryngii* var. *eryngii* grape pomace; PEM, *Pleurotus eryngii* var. *eryngii* mixture; W, water; E, absolute ethanol.

### Appendix A.2

**Table A2:** Total polyphenol content of mushroom extracts.

	Gallic Acid Equivalents (mg/mL Extract)					
	POS	POP	POM	PES	PEP	PEM
water (W)	0.256 ± 0.011 ^a^	0.113 ± 0.002 ^b^	0.124 ± 0.013 ^b^	0.070 ± 0.006 ^b^	0.085 ± 0.008 ^a^	0.071 ± 0.002 ^b^
abs. EtOH (E)	0.087 ± 0.006 ^a^	0.062 ± 0.002 ^b^	0.060 ± 0.008 ^b^	0.030 ± 0.003 ^c^	0.050 ± 0.004 ^a^	0.040 ± 0.002 ^b^

The results are expressed as means ± S.D. from at least three independent experiments. *p*-Values were determined using a one-way ANOVA followed by Tukey–Kramer post hoc test. Values with the same letter within a line are not significantly different at α = 0.05. Abbreviations: POS, *Pleurotus ostreatus* wheat straw; POP, *Pleurotus ostreatus* grape pomace; POM, *Pleurotus ostreatus* on mixture; PES, *Pleurotus eryngii* var. *eryngii* wheat straw; PEP, *Pleurotus eryngii* var. *eryngii* grape pomace; PEM, *Pleurotus eryngii* var. *eryngii* mixture; W, water; E, absolute ethanol.

### Appendix A.3

**Table A3:** Effects of different mushroom extracts on ferric reducing antioxidant power (FRAP).

	Ascorbic Acid Equivalents (mg/mL Extract)					
	POS	POP	POM	PES	PEP	PEM
water (W)	0.112 ± 0.006 ^a^	0.066 ± 0.004 ^c^	0.080 ± 0.015 ^b^	0.029 ± 0.002 ^c^	0.046 ± 0.003 ^a^	0.033 ± 0.008 ^b^
abs. EtOH (E)	0.034 ± 0.001 ^a^	0.028 ± 0.001 ^b^	0.027 ± 0.002 ^b^	0.016 ± 0.001 ^c^	0.025 ± 0.004 ^a^	0.020 ± 0.002 ^b^

The results are expressed as means ± S.D. from at least three independent experiments. *p*-Values were determined using a one-way ANOVA followed by Tukey–Kramer post hoc test. Values with the same letter within a line are not significantly different at α = 0.05. Abbreviations: POS, *Pleurotus ostreatus* wheat straw; POP, *Pleurotus ostreatus* grape pomace; POM, *Pleurotus ostreatus* mixture; PES, *Pleurotus eryngii* var. *eryngii* wheat straw; PEP, *Pleurotus eryngii* var. *eryngii* grape pomace; PEM, *Pleurotus eryngii* var. *eryngii* mixture; W, water; E, absolute ethanol.

### Appendix A.4

**Table A4:** Effects of different mushroom extracts on radical scavenging activity (DPPH).

	TROLOX Equivalents (mg/mL Extract)					
	POS	POP	POM	PES	PEP	PEM
water (W)	0.028 ± 0.006 ^c^	0.040 ± 0.001 ^a^	0.031 ± 0.002 ^b^	0.023 ± 0.003 ^a^	0.020 ± 0.001 ^b^	0.023 ± 0.001 ^a^
abs. EtOH (E)	0.023 ± 0.006 ^c^	0.039 ± 0.001 ^a^	0.035 ± 0.002 ^b^	0.010 ± 0.001 ^c^	0.024 ± 0.001 ^a^	0.018 ± 0.002 ^b^

The results are expressed as means ± S.D. from at least three independent experiments. *p*-Values were determined using a one-way ANOVA followed by Tukey–Kramer post hoc test. Values with the same letter within a line are not significantly different at α = 0.05. Abbreviations: POS, *Pleurotus ostreatus* wheat straw; POP, *Pleurotus ostreatus* grape pomace; POM, *Pleurotus ostreatus* mixture; PES, *Pleurotus eryngii* var. *eryngii* wheat straw; PEP, *Pleurotus eryngii* var. *eryngii* grape pomace; PEM, *Pleurotus eryngii* var. *eryngii* mixture; W, water; E, absolute ethanol.
